# Therapeutic Potential of Brassinosteroids in Biomedical and Clinical Research

**DOI:** 10.3390/biom10040572

**Published:** 2020-04-09

**Authors:** Sukhmeen Kaur Kohli, Abhay Bhardwaj, Vinay Bhardwaj, Anket Sharma, Namarta Kalia, Marco Landi, Renu Bhardwaj

**Affiliations:** 1 Plant Stress Physiology Lab, Department of Botanical and Environment Sciences, Guru Nanak Dev University, Amritsar 143005, Punjab, India; sukhmeenkohli@gmail.com (S.K.K.); anketsharma@gmail.com (A.S.); 2 Department of Bio-organic and Biological Chemistry, Kharkiv National Medical University, Kharkiv 61000, Ukraine; Abhaybhardwaj1995@gmail.com (A.B.); Vinaybhardwaj888@gmail.com (V.B.); 3 State Key Laboratory of Subtropical Silviculture, Zhejiang A&F University, Hangzhou 311300, China; 4 Department of Molecular Biology and Biochemistry, Guru Nanak Dev University, Amritsar 143005, Punjab, India; kalianamarta62@gmail.com; 5 Department of Agriculture, Food & Environment, University of Pisa, Via del Borghetto 80, 56124 Pisa, Italy; 6 Interdepartmental Research Center Nutrafood “Nutraceuticals and Food for Health”, University of Pisa, Via del Borghetto 80, 56124 Pisa, Italy

**Keywords:** brassinosteroids, anticancerous, antiviral, antibacterial, ecdysteroidal activities

## Abstract

Steroids are a pivotal class of hormones with a key role in growth modulation and signal transduction in multicellular organisms. Synthetic steroids are widely used to cure large array of viral, fungal, bacterial, and cancerous infections. Brassinosteroids (BRs) are a natural collection of phytosterols, which have structural similarity with animal steroids. BRs are dispersed universally throughout the plant kingdom. These plant steroids are well known to modulate a plethora of physiological responses in plants leading to improvement in quality as well as yield of food crops. Moreover, they have been found to play imperative role in stress-fortification against various stresses in plants. Over a decade, BRs have conquered worldwide interest due to their diverse biological activities in animal systems. Recent studies have indicated anticancerous, antiangiogenic, antiviral, antigenotoxic, antifungal, and antibacterial bioactivities of BRs in the animal test systems. BRs inhibit replication of viruses and induce cytotoxic effects on cancerous cell lines. Keeping in view the biological activities of BRs, this review is an attempt to update the information about prospects of BRs in biomedical and clinical application.

## 1. Introduction

In living systems, biochemical interaction among cells is achieved by chemical messengers that pass between them. These chemicals serve to coordinate the metabolic activities of various tissues and also allow individuals to adapt to changing environments and prepare them for reproduction. One of the major classes of chemical messengers is known as hormones. In animals, hormones are synthesized in specialized ductless glands and are carried by the circulatory system to other parts of the body. On the other hand, plants have the capability to synthesize hormones, which is rather widely scattered in cells. Although, the site of synthesis varies as the plant develops and the hormones may only be translocated relatively short distances [1,2]. Steroids, a crucial category of hormones, play an essential function in plant signaling responses and modulate a plethora of physiological and growth attributes. Several sex hormones, steroidal in nature, have also been reported in plants such as corticosteroids, estrone, progesterone, testosterone, and large number of analogues of these compounds [3,4]. Exogenous supplementation with these steroidal sex hormones results in significant modification of morphological and physiological responses in plants and animals [5,6,7].

Acetate is primary precursor for synthesis of cholesterol. It is postulated that three acetate molecules combine to form a single five carbon unit called isoprene. Cholesterol is an acyclization product of squalene, a 30-carbon polymer made up of six isoprene units. Squalene could readily be converted into cholesterol involving the synthesis of lanosterol through many steps. Cholesterol after a series of side chain cleavages and oxidations is converted to Delta-5-pregenolone which is further converted to progesterone by a dehydrogenase or to 17α-hydroxy-pregenolone by a specific 17-hydroxylase. Biosynthesis of steroids in plants is similar to that in animals. Different species are able to transform cholesterol to several steroids, for example, cholesterol is transformed to pregenolone by *Digitalis* species [8,9,10] and by *Haplopappusheterophyllus* [11]. The *Digitalis lanata* plants also transform sitosterol to progesterone [12]. Progesterone is then converted to corticosterioid by *D. lanata* [13]. The *Mallototuspaniculatus* plants alter cortisol to cortisone [14]. Similarly, observation revealed transformation of cholesterol and sitosterol, applied on plants, to a wide array of steroid hormones present in animals. The observations revealed that certain plants are able to synthesize Delta-4-3-oxo-steroids [15].

**Figure 1:**
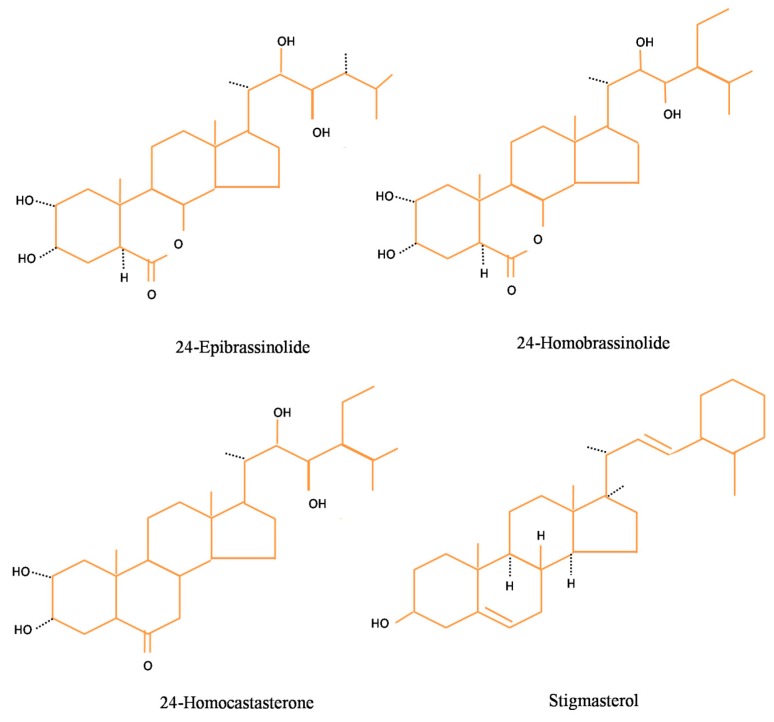
Structure of basic brassinosteroids.

A novel group of steroidal hormones was discovered in 1970, which had structural similarity with animal steroidal hormones. BRs are polyhydroxy steroidal hormones, first isolated by Grove et al. [16] from pollens of *Brassica napus* and were referred as brassinolide (BL). The second BR isolated was castasterone (CS) by Yakota et al. [17]. Since then, 70 different BRs (five conjugated and 65 un-conjugated) have been identified in various plant species [18,19]. The endogenous content of BRs varies from tissue to tissue as well as in micro-molar and nano-molar concentrations [20,21,22]. The immature seeds of plants and their pollens have been reported to have the highest concentration of BRs in them and are also dependent upon the age of plants [23,24]. Like their counterparts or similar analogue, BRs have a wide array of responses in various developmental and physiological functions of plants such as germination, regulation of gene expression, cellular and vegetative growth, differentiation of vasculature, root growth, apoptosis, homeostasis, and reproductive development [25,26,27,28]. BRs not only have an imperative role in plant development at the organ level but also at the cellular level. It has been reported to trigger fission and elongation of cell, activate protein and nucleic acid synthesis, elevate photosynthetic efficiency, structural changes in the cell wall, and modulation of permeability of the cell membrane [29]. Figure 1 depicts the structures of certain basic brassinosteroids viz. 24-EpiBL, 28-HomoBL, 28-Homocastasteone, and stigmasterol.

Moreover, BRs also play a dynamic role in plant acclimatization in response to varied stresses [30,31,32]. External supplementation of BRs triggers innate potential of plant to survive against stresses, specifically receding biotic stresses caused by variable pathogens including fungi, bacteria, and viruses [33,34,35]. Application of BRs has also been found to control cell proliferation and promote cell death of cancer cells in various mammals, including humans [36,37,38]. Beside anticancerous effects, they possess antiviral, antifungal, antibacterial, and ecdysteroidal properties. They have also been observed to heal cutaneous wounds by controlling inflammatory and re-epithelialization processes [39].

This review is an attempt to update the information about prospects of BRs in biomedical and clinical applications.

## 2. Dietary Sources of Phytosteroids in Plant

BRs are identified as one of the most widespread phytosterols detected in wide array of plant species belonging to different families [16]. They further include varied bioactive analogues viz. castasterone, brassinolide, typhasterol, 6-deoxocastasterone, and teasterone etc., being the most common [40,41,42]. Of all these, brassinolide and castosterone are comparatively highly distributed and have elevated bioactivities amongst the other BRs in varied plant species [43,44]. It is quite evident that due to their commercial availability, 24-EpiBL and 28-HomoBL, they are the most frequently studied compound [41]. The biological activities or biological potential of BRs is reliant on its structure with 2α, 3α-dihydroxy-7-oxa-6-ketone moiety. The contents of phytosterols in plants, specifically vegetable crops, have been reported by Han et al. [45]. They enlisted a plethora of sterols in mg/100 g of fresh weight (FW) of varied vegetable crops. The content of total phytosterols in onion bulbs, scallion, garlic, celery, cauliflower, cabbage, broccoli, rape, sweet pepper, and coriander leaves were 7.4, 22, 11.2, 14.2, 42.8, 13.6, 40.9, 10.2, 3.2, and 18.7 mg/100 g of FW, respectively. Furthermore they quantified total phytosterols in cucumber (7.3 mg/100 g of FW), zucchini (16.7 mg/100 g of FW), carrot (19.4 mg/100 g of FW), soybean (15.2 mg/100 g of FW), sweet potato (9.9 mg/100 g of FW), pea (53.7 mg/100 g of FW), potato (3.6 mg/100 g of FW), tomato (6.1 mg/100 g of FW), and ginger (15.0 mg/100 g of FW), respectively. Another report by Piironen et al. [46], revealed the amount of sterols in different vegetable crops including leeks (19.4 mg/100 g of FW), dill (32.5 mg/100 g of FW), swede cauliflower (31.0 mg/100 g of FW), brussels (37.0 mg/100 g of FW), broccoli (36.7 mg/100 g of FW), cucumber (78.1 mg/100 g of FW), carrot (15.3 mg/100 g of FW), lettuce (8.5 mg/100 g of FW), pea (29.7 mg/100 g of FW), tomato (7.4 mg/100 g of FW), and potato (5.1 mg/100g of FW).

## 3. Mechanism of Action of BRs

**Figure 2:**
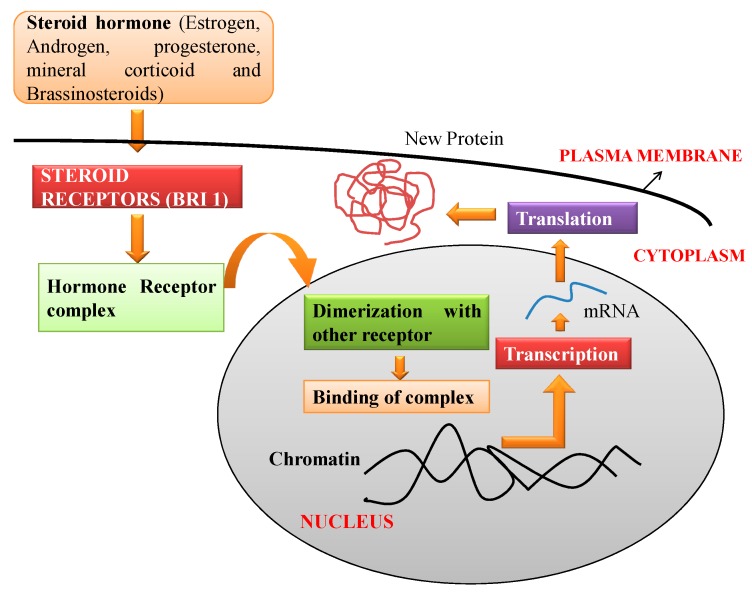
Schematic representation of molecular mechanism of brassinosteroid (BR) action in the animal cell.

In animal cells, the mechanistics of the steroids involves binding of hormone receptor proteins which are located either inside or on the cell membrane of the responsive cells. The steroid-receptor complex is able to enter the nucleus where it binds reversibly to specific sites on the chromatin of the cell nucleus. This interaction leads to the activation (or sometime inhibition) of the rate of the transcription of specific genes. The messenger RNA, which is synthesized, is translated into proteins [47]. Similarly, BR binds to receptor kinase i.e., BRI 1 (brassinosteroid-insensitive 1) present on the plasma membrane surface which results in initiation of BR signaling and genomic responses [48]. In the animal system, the steroids which are lipophilic in nature bind to the receptor present in the nucleus or cytosol which has diffused through the plasma membrane. Ligand complex formation results in conformational changes and dimerization with other receptors. This alteration stimulates the ligand-receptor complex to modify the DNA sequence which directly results in variation of gene expression [49]. Figure 2 is a schematic representation of molecular mechanism of BR action in the animal cell.

Rarova et al. [50] suggested a similarity between human steroids and BRs. They studied the interaction between BRs and human steroids using competitive binding assay and reporters assay. Competitive binding assay showed no evidence of interaction of 24-epibrassinolide (24-EpiBL) and 28-homocastasterone (28-homoCS) with Estrogen receptor α (ER-α) and Estrogen receptor β (ER-β), respectively. Modulation of *PR* (parthenogenesis) gene expression is directly proportional to concentration of 24-EpiBL and 28-homoCS as evident from Western blotting. There exists an independent or steroid-receptor dependent mode of action, in a similar manner as 2-methoxyestradiol binds to estrogen receptors. A report by Steigerova et al. [51] suggests that BR supplementation of MCF7 breast adeno carcinoma cells results in modulation of localization patterns of ER-α and ER-β. It was also noticed that there was a uniform and strong ER-α immune-nuclear labeling in MCF cells (untreated cells) and cytoplasmic speckles were revealed in MCF cells supplemented with 28-homoCS and 24-EpiBL. The ER- α and ER-β were predominantly present in control MCF-cells. Although, ER-β was relocated in the nuclei after treatment with 28-homoCS, however, it was present at the periphery of the nucleus after treatment with 24-EpiBL.

Furthermore, it was accompanied by lowered ERs levels after BR application [51]. It was further suggested that BRs hinder cell growth and different phases of cell cycle. There was an upregulation of cyclin-dependent kinases (CDKs) inhibitors i.e., p21^WAf1/Cip1^ and p27^kip1^. These inhibitors are reported to suppress the activities of cyclin/CDK complexes. It was further suggested that treatment with BRs also induces apoptosis in breast cancer cells (estrogen-sensitive and estrogen-insensitive). The induced apoptosis was affirmed by employing TUNEL staining, acridine orange, and staining with propidium iodide of both cancer cell lines i.e., MCF7 and MDA-MB-468. Although, the change in expression of apoptosis related proteins stimulated by BRs was different for different cell lines [51].

## 4. Therapeutic Role of BRs

Recent development in studies related to bioactivities of BRs in several animal models indicates their significant potential in therapeutic applications. They are suggested as anticancerous, antiproliferative, antiangiogenesis, antiviral, antigenotoxic, antifungal, and antibacterial, and display anti-chloesterolemic activities, synthetic medication [36,52,53,54] which are briefed below:

### 4.1. Anticancerous/Antiproliferative Activities

Sterols with oxygenated side chains have been procured from several plants and marine organisms which are toxic to mammalian cancerous cells [55]. BRs are known to have therapeutic characteristics against cancer development and show potential for developing new anticancer drugs [36,56,57,58]. Several studies further reported that a BRs analogue with similar oxygenated side chain had similar activities as that of sterol [59,60]. Cancer cells have distinctiveness of indefinite proliferation and not undergoing apoptosis normally. BRs can stimulate obligatory induction of necessary responses required for inhibition of growth and stimulation of apoptosis by interfering with the cell cycle [36]. Various BRs have been studied for anticancer bioactivities in various cell lines i.e., CEM (T-lymphoblastic leukemia), A-549 (lung carcinoma), MCF-7 (breast carcinoma), RPMI 8226 (multiple myeloma), HeLa (cervical carcinoma), MDA-MB-468 (breast carcinoma), and LNCaP (prostate cancer) etc. All these cell lines were observed for viability test after six serial 4-fold dilutions for 72 h. IC_50_ value of BRs as observed from Calcein AM assay gives an idea of their potential to be used as anticancer drugs [36].

As reported by Malikova et al. [36], 28-homoCS, CS and its homologue *22S,23S*-28-homocastasterone showed maximum cytotoxicity for cancer cell lines and BRs showed a different kind of response in different cells as no viability loss was observed in the fibroblast cells. The effect of BRs (28-homocCS and 24-EpiBL) was studied on viability of these human cancer cell lines (CEM and RPMI 8226). It was reported that both (28-homoCS and 24-EpiBL) reduced the viability of CEM and RPMI 8226 in a dose dependent manner even at micro molar concentration and 28-homocastasterone was more active and potent for CEM cells [36]. These two BRs were also analyzed for the anticancer effects in the breast cancer cell lines MDA-MB-468 (estrogen receptor-α-negative) and MCF-7 (estrogen receptor-α-positive), and prostate cancer cell lines DU-145 (androgen-insensitive), and LNCaP (androgen-sensitive). The androgen and estrogen (sensitive and insensitive) cell lines behave diversely when exposed to the BRs. Most of prostate cancer cells have both sensitive and insensitive types of cells and are required for the elimination of cancer [61,62]. It has been reported that hormone responsive cell lines could be more efficiently treated with BRs. Hence, the research has been directed towards the probable inflection of steroid receptor-mediated response of BRs and development in many test models applied for advancement of new BR derived anticancerous drugs [33]. 28-homoCS and 24-EpiBL influence proliferation, survival, and cell cycle of breast cancer cell lines (DU-145 and LNCaP). Cell growth is inhibited by both BRs in a dose dependent manner in cancerous cell lines. Analysis by flow cytometry showed that treatment of BR inhibited MDA-MB-468, MCF-7, and LNCaP cells in G_1_ phase and stimulates cell death in LNCaP, MDA-MB-468, and minutely in DU-145 cells resulting in reduction of cell percentage in S-phase. In response to application of antiestrogen, MCF-7 cancerous cell lines suppressed cell proliferation resulting in reduced quantity of cells synthesizing DNA (S phase) and shifts to G_o_/G_1_ phase [63]. It was reported by Oklestkova et al. [37] that BRs suppress the growth of varied cancerous cell lines at µM levels without altering normal cell growth. BRs are applied to cure disorders, and a few of them involve proliferation of cells and include Huntington disease, Alzheimer disease, sexual differentiation disorders, cancer, and steroid induced osteoporosis, hyperadrenocorticism linked with excess of sex steroids, steroid induced cataract, glucocorticoid insensitive asthma, and P450 oxidoreductase deficiency. BRs influences differentiation, proliferation, survival, and expression of certain cell cycle associated proteins in cancerous cell lines [64].

The mechanism of antiproliferative activity of 28-homoCS and 24-EpiBL was studied by Steigerova et al. [51,65] in prostate cancer cell lines, DU-145 and LNCaP (hormone insensitive and sensitive). Various techniques like flow cytometry, TUNEL DNA ladder Test, Western blotting, and immunofluorescence were used to study the effect of these BRs on prostate cancer cells. Repression of cell growth and blockage at G_1_ phase along with decline in CDK4/6, cyclin D1, and pRb expression was recorded in LNCaP cells. However, in DU-145 cells a high proportion of cells were observed in the G_2_/M phase along with reduction of expression cyclins A and B_1_. Apoptosis initiated by BRs was observed with an increase in the subG_1_ fractions in DU-145 and LNCaP cell lines in TUNEL staining and identification of DNA (low molecular weight) recognized by bands visible in Western blotting.

BRs are recommended to cure the harmful effects of hyper proliferation in mammalian cells in vivo as well as in vitro, especially to cure hyper proliferative disorders. 28-homoCS also reduces the expression of p53, p27, p21, Bcl-2 family anti-apoptotic proteins, cyclins, and ER-alpha (estrogen receptor α) [33]. A 24-EpiBL treatment (10^−6^ to 10^−9^ mol L^−1^) modified growth properties of mouse hybridoma cultured under nutritional stress due to growth in either standard medium (serum free) or 30% diluted medium. Enhancement of mitochondrial membrane potential, number of cells in G_0_/G_1_ phase and S phase, and reduction of intracellular antibody have been reported by 24-EpiBL treatment.

The anticancer activity of EBR against prostate cancer cells by inducing apoptosis in androgen sensitive cells (LNCaP) was investigated using SILAC (stable-isotope labeling by amino acids in cell culture). The cancer cells, prior to 24-EpiBL treatment, were quantified for proteins and it showed that expression of calreticulin (CALR) an unfolded protein response (UPR) chaperone protein was under expressed and initiated the CHOP (an antibody against ER stress) translocation from the cytoplasm to nucleus. This CHOP translocation activates the caspase-9 and caspase-3 enzymes. Co-application of 24-EpiBL with rapamycin (a well-known translational inhibitor) inhibits the cell death and PARP cleavage in LNCaP cells suggesting that 24-EpiBL could induce ER stress in these cells [66]. However, in another study 24-EpiBL treatment has been found to enhance the cell death in prostate cancerous cell lines (LNCap and DU 145) by increasing the expression of PAO (polyamine oxidase) and SSAT (spermine N1-acetyltransferase) enzymes [66]. Brassinolide has been observed to promote apoptosis of human prostate cancer cell line, PC-3, by enhancing caspase-3 activity and downregulating the expression of Bcl-2 (an anti-apoptotic protein) [67]. Application of 24-EpiBL in colon carcinoma cells (HT-29 and HCT 116) have been found to upregulate Foxo3a (Forkhead/Winged Helix Box Class O) and protein tyrokinase Src p38, after the activation of PI3K/AKT, hence leading to mitochondria-regulated cell death in colon cancer cells [68]. Antiangiogenic property of the BRs and its derivative (cholestanon) was checked in human micro vascular endothelial cells (HMEC-1) and human umbilical vein endothelial cells (HUVEC). It was observed that 24-EpiBL and 28-HomoCS inhibited proliferation of HMEC-1 cells in a concentration dependent manner and also inhibited migration of HUVEC cells and cholestogen strongly decreased the cell adhesion, affecting antiproliferative activity of the cells. The weakest opponent of estrogen-receptor-α (ERα) was found to be 24-EpiBL. The results showed that BRs and its analog BR4848 can suppress in vitro angiogenesis of primary endothelial cells and inhibit angiogenesis and prevent the development of metastasis [50,69].

### 4.2. Antiviral Activities

The primary facet of the defensive role of BRs is associated with their capability to provide resistance against viruses [70,71]. The first line of inner resistance to stress is swiftly induced by host recognition by presence of conserved structural motifs which is entirely articulated by pathogens. These motifs are recognized as PAMPs and are discharged by the host throughout pathogen attack or DAMPs i.e., damage-associated molecular patterns discharged from wound [72]. The detection of various DAMPs/PAMPs by particular sensors present on the cell surface, known as PRRs, stimulates a refined defense signaling mechanism which hampers a wide range of potential pathogens such as viruses, bacteria, oomycetes, and fungi. PRRs are selected by receptor-like proteins and receptor-like kinases situated on the surface of the cell. Receptor-like kinases/proteins require a co-receptor in order to generate an activated complex to start signaling. BRASSINOSTEROID INSENSITIVE (BRI1)-related kinase BAK1 is the best classified co-receptor of PTI which produces active signaling complexes with receptor-like kinases as well as receptor-like proteins after PAMP recognition through PRRs [73]. Interaction of PRRs and LRR-RLK BRI1 with BAKI is directly proportional to availability of ligands. BAK1 is imperative for plant immunity under RNA virus infestation. *bak1-4* and *bak1-5* mutants were reported to have higher levels of TCV, ORMV, and TMV accumulation when compared to wild plants [74]. BR treatment significantly decreased virus infection and enhanced the crop yield by 56%. Tobacco plants treated with brassinolide (BL) induced resistance against TMV [75]. BAK1 or BKK1 are essential components in controlling Turnip crinkle virus infection in *Arabidopsis thaliana* [76]. BRs supplementation increased antioxidative enzyme activity, regulated the defense associated gene expression, and reduced photosystem deterioration in *A. thaliana* under cucumber mosaic virus infection [77].

**Figure 3:**
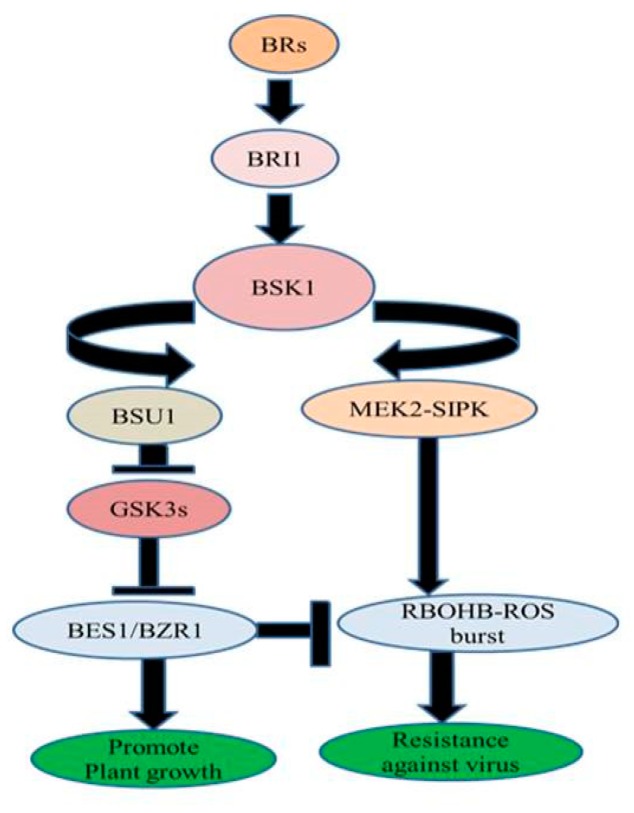
BR-regulated virus resistance.

In *Nicotiana benthamiana*, exogenous treatment of BRs increased resistance against TMV through MEK2 (MAPKK)-SIPK (salicylic acid-induced protein kinase) and RBOHB (respiratory burst oxidase homolog protein B)-dependent ROS burst. BES1/BZR1 suppressed RBOHB-dependent ROS generation and function as an essential mediator in BR signaling between growth and immunity. When the activated form of BZR1/BES1 is present in low concentration, RBOHB-dependent ROS burst generation is regulated by MEK2-SIPK signaling network which might have offered resistance to TMV. On the other hand, when the active form of BZR1/BES1 is present in elevated concentrations subsequent enhanced BR signaling might have inhibited RBOHB-dependent ROS formation by BZR1/BES1 and stimulated plant development (Figure 3) [78].

Antiviral properties of BRs in response DNA and RNA viruses are tabulated in Table 1.

**Figure 4:**
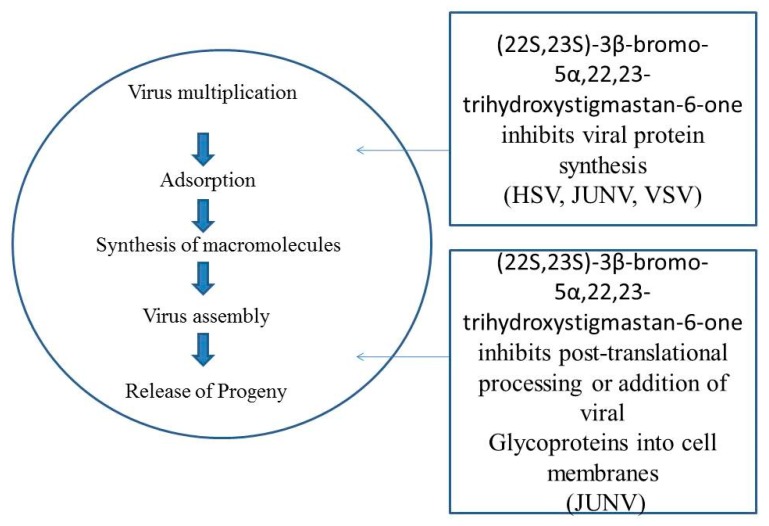
[22S, 23S]-3β-bromo-5α,22,23-trihydroxystigmastan-6-one targets possible sites in the virus multiplication cycle.

It has been elucidated that various synthetic analogues have antiviral activity against measles (MV), polio virus (PV), arena viruses, vesicular stomatitis virus (VSV), and herpes simplex type 1 and 2 (HSV-1 and HSV-2) [52,53,80]. It has been illustrated that (22S,23S)-3β-bromo-5α,22,23-trihydroxystigmastan-6-one primarily affects the late phase of the growth of virus. The inhibitory action did not target the adsorption processes, internalization, and premature production of RNA of the virus. Results showed that this compound has a deleterious effect on protein and mature particle synthesis of viruses [81]. In Vero cells, the antiviral property of compound [22S,23S]-3β-bromo-5α,22,23-trihydroxystigmastan-6-one was evaluated against Junin virus (JV). It was demonstrated that (22S, 23S)-3β-bromo-5α,22,23-trihydroxystigmastan-6-one principally influences the premature virus growth phase. Virus adsorption processes and internal development was not the targeted of the enhanced activity of the inhibitors. This compound has a detrimental effect on RNA replication of the virus by prohibiting the formation of full length antigenomic RNA. (22S,23S)-3β-bromo-5α,22,23-trihydroxystigmastan-6-one has productive results depending on infestation by JV, an elevated level of JV inhibition, and blending activities of lately formed viral glycoproteins [82] (Figure 4). Treatment of BR enhanced the accumulation of nitric oxide and reduced the accumulation of cucumber mosaic virus (CMV) in *A. thaliana*. Results showed that nitric oxide is involved in BR-stimulated virus resistance in plants [83].

### 4.3. Antiherpetic Activities

Herpes viruses are ubiquitously present in animal kingdom and have evolved over a period of million years with their host. Presently, eight different human herpes viruses have been recognized out of which herpes simplex (HSV) is omnipresent. Herpes infection is characterized by their aptitude to cause lifelong infections [84].

HSV infections are recurrent mucocutaneous in nature, they are severely painful infections, and are of social concern, although they are not the most serious infections. HSV-1 infection not only produces lesions on the mucous membrane that are ulcerative in nature, they also stimulate an immunopathology when the infection is caused in the eyes. Following are stages of infection caused by HSV-1 in the eyes: (i) inflammatory response is triggered, (ii) irreparable damage of the cornea, and (iii) temporary loss of host vision or even permanent blindness [85,86]. As HSV is considered to be a long-lasting infection, its recurrence is considered as significant medical burden. Moreover, HSV infections result in other severe complications including pneumonia, hepatitis, keratitis, encephalitis, and esophagitis [87]. To date, success with development of an HSV vaccine is very preliminary. Few drugs such as acyclovir [ACV] were used in the past three decades for treatment of HSV infection. Among the most infrequent but severely life-changing diseases sourced from HSV is encephalitis (HSVE) in adults and newborns [88]. Similar to other HSV treatments, acyclovir is effective in treating HSVE by lowering the mortality but was found to be less effective in eradicating morbidity. It was suggested by Kamei et al. [89], that the combined treatment with acyclovir and corticosteroids gave much more effective results than acyclovir alone, when given to adult patients with HSVE infection. Corticosteroids counter HSVE infection by inhibiting pro-inflammation of cytokines such as IL-6 (Interleukin-6) [90].

Brassinosteroids (BRs) are considered as a novel candidate of steroids that play a crucial role in antiviral activities in the recovery of patients suffering with herpetic and viral diseases [91]. Four analogues of 28-homocastasterone such as (22R,23R,24S)-3β-acetoxy-22, 23-dihydroxy-5α-stigmastan-6-one (1), (22R,23R,24S)-3β-bromo-22,23-dihydroxy-5α-stigmastan-6-one (2), (22R,23R,24S)-3β-acetoxy-5, 22, 23-trihydroxy-5α-stigmastan-6-one (3), and (22R,23R,24S) 3β-bromo-5,22,23-trihydroxy-5α-stigmastan- 6-one [4] have been formed via an artificial pathway dependent upon regioselective ∆^5^ epoxidation and characterization was done from stigmasterol. Compounds 1 and 2 having a 5αH moiety were synthesized by a reductive opening of the 5β, 6β epoxy precursor, and compounds 3 and 4 having 5αOH moiety were attained through hydrolytic opening of a mixture of 5α, 6α and 5β, 6β epoxy precursors. BRs were taken as standards during the tests performed with traditional auxin-like bioassay. The results revealed that these compounds act as an inhibitor of DNA (HSV-1) virus replication [92]. Another observation by Wachmann et al. [52] suggested that BR analogue (22*S*,23*S*)-3β-bromo-5α,22,23-trihydroxystigmastan-6-one hampered the herpes simplex virus [HSV] type 1 replication in Vero cells by affecting the later stages of virus multiplication. By immunofluorescence studies it has been revealed that this analogue inhibited the expression of HSV antigen and reduced the production of HSV late protein [52,93].

In the IOBA-NHC cell line, BR analogues showed their effect in a dose dependent manner when supplemented subsequent to infection which demonstrated no cytotoxicity and averted the multiplication of HSV-1. The analogues 1, 2, and 3 have been administrated to the eyes of mice, and it has elucidated that 1, 2, and 3 days after infection postponed and decreased the frequency of HSK such as vascularization, necrosis, and inflammation in comparison to untreated tainted mice [94]. Moreover, further studies suggested that these stigmasterol derivatives also inhibit synthesis of severe tumor necrosis factors (TNF-α) in a lipopolysaccharide stimulated (LPS) murine macrophage cell line. These compounds stimulated discharge of interleukin-6 (IL-6) and TNF-α in HSV-1 infested cells; which exerted an in vitro immune-regulatory effect [95]. These macrophages are ubiquitously present in the cell and have distinct functional abilities such as phagocytosis, microbial apoptosis, surface adsorption, and motility [96]. These compounds were applied in a mouse system with HSV-1 infection having induced ocular disease and were monitored in distinct treatment modalities [97]. This treatment was found to lower the severity and incidence of lesions caused by HSV-1.

Few multifunctional stigmasterol derivatives have been recognized to have a very wide array of action in in vitro antiviral activities. These synthetic compounds play multiple roles including: (i) inhibition of multiplication of RNA viruses and (ii) restraining HSV-1 infections [80,81]. EBL is recognized as a potential compound in the cure of human immunodeficiency virus (HIV) infection and other associated conditions in addition to help in the reduction of blood serum cholesterol for remedial and prophylactic applications in medicine and specialized nutrition. The applications of this steroidal plant hormone helped in recovering blood cholesterol and gave a new direction in the treatment of infections caused by HIV [98,99]. Antiherpetic activity of synthetic analogues of BRs such as (22*S*,23*S*)-3β-bromo-5α, 22, 23-trihydroxystigmastan-6-one (1), (22*S*,23*S*)-5α-fluoro-3β-22, 23-trihydroxystigmastan-6-one (2), (22*S*, 23*S*)-3β,5α, 22, 23-tetrahydroxy-stigmastan-6-one (3) has been studied in both human conjunctive cell lines (IOBA-NHC) and murine herpetic stromal keratitis (HSK). Human HSK is not cured through antiviral drugs although the symptoms might be ameliorated via certain immunosuppressing agents such as cyclosporine and corticosteroids. Development of therapeutic vaccinations is a proficient technique to lessen the effect of persistent nature of HSV ocular disease, possibly by suppressing the reactivation [85,100]. Another important example of inhibition of HSV-1 replication in human is application of two stigmasterol derivatives including (22S,23S)-22, 23-dihydroxystigmast-4-en-3-one and (22S,23S)-3β-bromo-5α, 22, 23-trihydroxystigmastan-6-one [95,101].

### 4.4. Antifungal and Antibacterial Activities

The prospect of BRs stimulating plant defense against fungal infestation has been reported in various experimental evidence [102]. BRs have been proven more protective than several fungicides. The mechanism by which BR increased resistance against this fungus is not due to pathogenesis related (PR) gene expression but due to SAR (systemic acquired resistance) inducers which activated immuno- protective responses within plants [75]. It has been reported that potato plants when treated with BRs showed lesser infection by *Phytophthora infestans*. This enhanced resistance in BR-applied potato tubers is attributed to stimulated levels of ethylene and abscisic acid along with existence of terpenoids and phenolics [23]. Moreover, it was investigated that 24-EpiBL, when exogenously applied to barley plants, reduced the fungal infection of leaves caused by *Helminthosporium teres* as well as increased the yield of the crop [103]. Additionally, it was studied that aqueous seed extracts of *Lychnisviscaria* containing BRs resulted in improved resistance of tomato, tobacco, and cucumber plants against fungal pathogen i.e., *Phytophthora infestans* and *Botrytis cinerea.* BRs induced peroxidases and altered apoplastic PR-proteins indicated activation of plant defense responses against fungal attack [104]. Another study was done in which 24-EpiBL when applied exogenously to tomato plants decreased the symptoms of *Verticillium dahlia* [25]. Additionally, it was demonstrated that BRs enhanced resistance against fungal pathogen *Oidium* in tobacco plants [75]. Moreover, it also stimulated the immune response of rice against blast disease caused by *Magnaporthegrisea* fungi. Furthermore, 24-EpiBL also ameliorated the toxic effects of *Verticillium dahliae* on cotton callus growth when applied exogenously [105]. It was however suggested that Vd toxin (*Verticillium dahliae*) caused wilting in plants which was reduced by the application of 24-EpiBL, demonstrating its role in mediating tolerance against *V. dahliae* in cotton [106]. Similar studies were reported by Zhua et al. [107] in jujibe fruits infested by blue mold rot caused by *Penicillium expansum* by exogenous application of BRs. They investigated that BRs when applied exogenously at 5 µM concentration suppressed the rot and increased the defensive enzymatic activities like superoxide dismutase, polyphenoloxidase, phenylalanine ammonia lyase, and catalase. Moreover, they suggested that BRs reduced *P. expansum* via inducing resistance in fruit along with delayed senescence [107]. 24 EpiBL- treated barley plants reduced the extremity of *Fusarium* head blight disease (FHB) induced by *Fusarium culmorum* and minimized the grain weight loss associated with FHB. 24-EpiBL showed 28% and 35% declined symptoms of disease in Lux and Akashinriki varieties of barley [108]. However, the steroleosin gene was found to be upregulated in 24-EpiBL treated plants during *Fusarium* infection and this gene is responsible for signal transduction in plants regulated by sterols [109]. Additionally, studies suggested the protective role of BRs against fungi in cucumber by increase in the enzymatic activities of polyphenoloxidases and peroxidases contributing majorly towards BR- enhanced disease resistance in cucumber [110]. Furthermore, brassinolide application, in post harvesting period extended the dormancy period of *Solanum tuberosum* as well as increased its resistance towards *P. infestans* [111].

It has been further reported that 1,2,4-triazole derivatives namely 1-[4-phenoxymethyl-2-phenyl-(1,3)dioxolan-2-ylmethyl]-1 H-1,2,4-triazole was highly active against *Magnaportheoryzae*. Its antifungal property was demonstrated in vitro during growth inhibition of mycelia [112]. Moreover, the key enzymes in the biosynthesis of brassinosteroids have been evaluated to study their role against *Magnaportheoryzae* [causing rice blast disease]. It was found that the compound 2RS, 4RS-1-[4-chlorophenyl-(2-methylphenoxy)-ethyl]-1,3-dioxolan-2-yl-methyl]-1H-1,2,4-triazole was a potent antifungal agent against *Magnaportheoryzae* [113]. Another study reported by Kim et al. [114] found that 24-epibrassinolide (EBL) reduced *Fusarium* wilt in cucumber and enhanced their antioxidant and phenolic levels in roots. Therefore, EBL minimized fungal initiated ROS and increased resistance towards *Fusarium* wilt in cucumber roots. It has been observed that *Verticillium dahlia* causes huge loss of cotton yield, where brassinosteroids were known to play functional role in providing resistance against this fungus. Exogenous treatment of brassinolide along with inoculation of *V. dahliae* in cotton seedlings activated the brassinosteroid signaling pathway that conferred resistance to these plants compared to that of control plants which demonstrated the positive role of BRs as antifungal agent [115]. Similarly, BRs (epibrassinolide) when applied hydroponically, enhanced resistance against the powdery mildew fungus *Oidium* sp. in *Hordeum vulgare* [116]. Additionally, media augmentation of brassinolide in *Oryza sativa* prevented the infestation related to root oomycete *Pythium graminicola* as reported by Vleesschauwer et al. [117].

Another aspect of the defensive facet of BRs is its antibacterial action. BL, one of the important brassinosteroids, has been reported to play a vital function in inducing disease resistance in plants. BL stimulated resistance in plants is distinctive from wound-inducible disease resistance and systemic acquired resistance (SAR) by not inducing or activating PR gene expression [31,75]. It was found by Nakashita et al. [75] that leaves of wild type of tobacco plants injected with bacterial pathogen *Pseudomonas syringae* pv. tabaci (*Pst*)when given treatment of BL result in significantly reduced lesion size compared to untreated plants. Similarly, *Xanthomonas oryzae* infected plant that was treated with BL resulted in elevation of defense action against the bacterial infection [75]. Similarly, Skoczowski et al. [118] revealed a protective role of BR27 in *Brassica napus* L. cotyledon when exposed with *Pseudomonas* infection. Intensity of brassinosteroid mediated disease resistance (BDR) varies with environmental factors especially light, temperature, humidity, etc. BRs play an important role in photo-morphogenesis [119] which shows the possibility of light signaling to induce BL-induced resistance [75].

### 4.5. Anti-Inflammatory Activities

Inflammation is a biological phenomenon that results in response to wounds, auto-immune diseases, infection, or allergy, etc. [120]. Inflammation of the particular tissue can be identified by heat, edema, redness, and pain. Excess of inflammation leads to wide array of diseases like inflammatory bowel diseases, allergic conjunctivitis, asthma, Crohn’s disease, allergic rhinitis, and chronic sinusitis [121]. The plants produce vast quantities of metabolites such as alkaloids, steroids, saponins, flavonoids, and many more with therapeutic value. There is a broad spectrum of plant steroids such as brassinosteroids, cardinolides, cucurbitacins, eclysteroids, with anolides, etc. known to possess antitumor, immunosuppressive, hepatoprotective, antiviral, and specifically anti-inflammatory properties [121]. Analogs of synthetic brassinosteroids including stigmastane and androstane derivatives are well known immunomodulating agents which also act as anti-inflammatory steroids [122]. BR analogs such as (22S,23S)-3β-bromo-5α, 22,23-trihydroxystigmastan-6-one, (22S,23S)-3β-5α, 22,23-tetrahydroxystigmastan-6-one and (22S,23S)-5α-fluoro-3β, 22,23-tetrahydroxystigmastan-6-one act against HSV-1 multiplication and exhibited anti-inflammation potential in vero cells [52]. It was found that after giving treatment with these compounds, inflammation began to decrease within three consecutive days.

Moreover, synthetic analog (22S,23S)-22,23-dihydroxystigmast-4-en-3-one has structural similarity with dexamethasone (DEX) which also showed anti-inflammatory properties in a HSK model [93]. This novel molecule (22S,23S)-22,23-dihydroxystigmast-4-en-3-one was synthesized from BRs in human conjunctiva cell line (IOBA-NHC) and a herpetic stromal keratitis (HSK) model in mice. It was found that this compound when administered in vitro exhibited reduction in inflammation as well as herpetic activities. Under in vivo conditions, anti HSV-1 activity was not so prominent, however, (22S,23S)-22,23-dihydroxystigmast-4-en-3-one improved HSK signs, revealing its imperative role in improving stromal inflammation [94]. A few other BR compounds such as oleanolic acid, ursolic acid, and (23R)-2α,3β,23,28-tetrahydroxy-14,15-dehydrocampestrol have been identified as anti-inflammatory agents. The hairy root line LRT7.31 acquired by infecting *Lopeziaracemosa* with *Agrobacterium rhiogenes* was reported to contain these BR analogs and possess anti-inflammatory activities [123]. In a murine model, wound healing on application of (22S,23S)-homobrassinolide gel accelerated wound repair process by enhancing the anti-inflammatory activity and re-epithelialization of skin. This was in response to increase in Akt signaling around the wound and promoting in vitro fibroblast migration to the inflamed area [124,125]. EBL has also been reported to be used for treatment of rough skins and has anti-wrinkle properties due to increased collagen formation in both epidermal cells and human dermal fibroblast [126].

Additionally, this compound also displayed anti-inflammatory activity and anti-adenoviral activity in adenoviral queratoconjunctivitis (ADV-QC) and human conjunctival cell line (IOBA-NHC) in an experimental model in rabbits under in vivo and in vitro conditions [127]. Furthermore, these stigmastane analogs (22S,23S)-22,23-dihydroxystigmast-4-en-3-one and (22S,23S)-22,23-dihydroxystigmasta-1,4-dien-3-one enhanced immuno-modulating activity and improved HSV-1 induced occular disease in mice [95]. It was suggested that these two compounds suppressed the replication of HSV-1 in nervous cells and its proliferation in human epithelial cells. Cytokine levels were determined after treatment with these compounds which reflected decreased levels of IL-6 and IFN-α in HSV-1 infected-Neuro-2a cells that demonstrated anti-inflammatory activities within them [84]. Reports by Michelini et al. [54] suggested that this compound reduced cytokine production in LPS activated macrophages and enhanced pro-inflammatory cytokine production in HSV-1 infected epithelial cells of mice cells. Additionally, the pro-inflammatory effect of this compound evoked an innate immune response at the site of infection. Moreover, overexpression of IL-10 and *Socs2* genes in LPS-cells was also observed in the presence of this compound. Socs2 acts as an anti-inflammatory agent that hinders NK-αB and JAK-STAT networks that further inhibit the discharge of many inflammatory cytokines such as IL-8, IL-6, IL-10, and TNF-α [128]. It was further investigated that 28-homobrassinolide acted as potential curative agent against leukotriene synthesis in 2H3 cells. Cytotoxic studies were done to observe the effect of 28-homobrassinolide on cell inhibition where no response of 28-homobrassinolide was found. However, Leukotriene C4 (LTC-4) and Leukotriene B4 (LTB-4) levels were stimulated which on treatment with 28-homobrassinolide was declined to some extent. Along with this, phospholipase A2 (PLA2) expression and Ca^2+^ concentration were drastically lowered on application of 28-homobrassinolide. All these observations showed the therapeutic role of 28-homobrassinolide in reducing inflammation by inhibition of leukotriene synthesis in 2H3 cells [129]. A study was conducted to observe the effect of phytosterol esters (PSEs) on non-alcoholic fatty liver disease (NAFLD) on rats in which it was found that PSE reduced the concentration of total cholesterol (TC), triglycerides (TGs), low-density lipoprotein cholesterol (LDL-C), and fatty acids. In addition, hepatic inflammatory stress was decreased through cytokine inhibition (IL-10, TGF-β, IL-6) and oxidative stress was reduced via antioxidative enzymes and reduced MDA contents. A CRP factor, recognized as cardiovascular disease (CVD) risk factor, was enhanced in NAFLD disease which upon PSE treatment was reduced which confirmed the anti-inflammatory potential of PSEs [130]. It was found that brassinosteroid keto isoform homocastasterone exhibited antiglycemic activity in diabetic rats and also improved RBC, WBC, platelets, hemoglobin levels, and reduced cell damage and inflammatory disorders such as inflammatory bowel disease, allergic reactions, and leukemia or myeloproliferative neoplasms in humans [131].

### 4.6. Antiangiogenic Activities and Antigenotoxic

The physiological phenomenon of development of novel blood vessels from previously formed micro vessels is termed as angiogenesis [132]. These are required to meet the demand of oxygen [133] and nutrients supply in animals for their organ growth as well as solid tumors, wound healing, in fetal and embryonal development, placenta formation, and for metastasis [134]. Angiogenesis is a highly regulated process in which endothelial cells are a major component of its tissues [135], which are targeted for antiangiogenic therapy. Tumor vessels have a deformed structure and size which makes them morphologically and functionally different from normal cell capillaries [136]. These cells are genetically unwavering, homogeneous, and also possess a low mutation rate making them unable to acquire drug resistance results due to its easy availability to antiangiogenic agents [134].

Tumor angiogenesis is targeted by using novel drugs which inhibits proteolytic enzymes, inhibits endothelial cell proliferation, collapsing of extracellular matrix of capillaries, and apoptosis of endothelial of the cancer cells [137]. Various natural steroidal compounds which have been reported to reduce angiogenesis are progestin, glucocorticoids, medroxy-progestrone acetate, and 2-methoxyestradiol [138]. Some natural BRs possess antiangiogenic activity by affecting endothelial cells [50] which includes 24-EpiBL and 28-homoCS along with two synthetic analogues of BRs (BR4848 and Cholestanon) in a dose dependent manner which restricts the proliferation of human microvascular endothelial cells (HMEC-1) as well as decreases the relocation of human umbilical vein endothelial cells (HUVEC) [134]. A BR analog form from (22S,23S)-22,23-dihydroxystigmast-4-en-3-one and (22S,23S)-22,23-dihydroxystigmasta-1,4-diene-3-one also exhibit antiangiogenic properties against solid tumors including breast cancer, skin cancer, and ovarian cancer [136].

**Figure 5:**
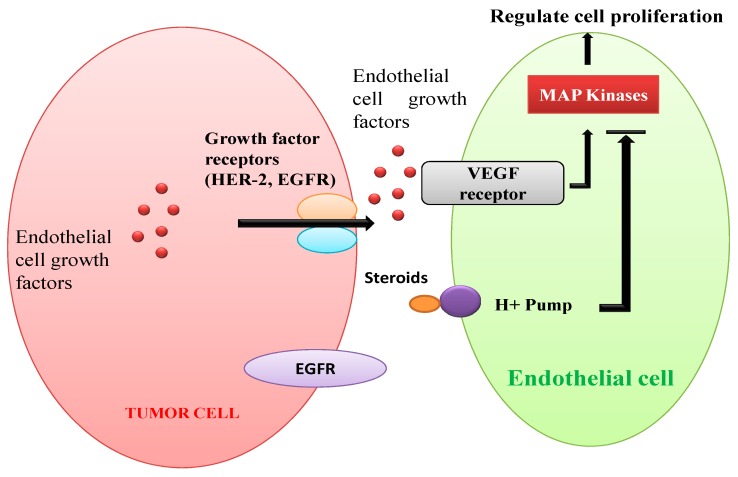
Schematic representation of angiogenesis in human solid tumors.

Figure 5 demonstrates angiogenesis in human solid tumors. Vascular endothelial cell growth factor (VEGF) receptors are stimulated by endothelial cell growth factors secreted by VEGFs (e.g., EGFR, HER-2) [138,139]. Regulation of cytoskeleton and cell proliferation of blood vessels through the MAPK pathway is stimulated by both peptides and steroids [138,140]. Squalamine, a steroidal compound is recognized to have strong antiangiogenic properties. It might be due to inactivation of MAP kinases present in the endothelial cells of blood by interacting with calmodulin, cell surface proton pumps, or other signaling pathways [141,142].

Moreover, natural BRs cause slight inhibition of tube formation whereas BR4848 blocks the G2/M phase of the cell cycle [143]. Further study by Michelini et al. [144] shows (22S,23S)-22,23-dihydroxystigmast-4-en-3-one (Compound 1) and (22S,23S)-3β-bromo 5α,22,23-trihydroxystigmastan-6-one (Compound 2), two synthetic stigmasterol derivatives, retard cell migration in HUVEC and capillary tube-like structure formation. Compound 1 prevents angiogenesis in vivo and in vitro, reduces the VEGF expression and also corneal neovascularization when applied during herpetic stromal keratitis (HSK). However, Hoffmannova [145] concluded BR as inhibitor of angiogenesis in primary human endothelial cells and its metastasis and also promotes apoptosis. Antiangiogenic properties of BRs alongside their anticancerous activity have become imperative for the advancement in manufacturing of novel anticancerous drugs [50]. A recent study done by Rarova et al. [69] suggested an antiangiogenic role of BR analog 2α,3α-dihydro-6-oxo-5α androstan-17β-yl-N-[tert-butoxycarbonyl]-D, L-valinate[BR4848] by inhibiting Akt phosphorylation, Erk 1 and focal adhesion kinase. However, they also suggested cytokine IL-6 as an activator of epithelial cell multiplication and migration may also control antiangiogenic properties of BR4848.

BRs show antigenotoxic potential in different plants when applied exogenously. The effect of 24-EpiBL on genetic structure and various cell processes in barley was studied by Khrustaleva et al. [146]. 24-EpiBL improves viability of cells and fertility rate but no significant and visible effect was observed in terms of aberrations. However, when applied along with nitro urea, decline in aberrations by double amount were observed in anaphase and tetrad phase. The mutagenic studies using Ames assay carried at the Scientific Research Center of Toxicologic and Hygienic Regulation of Bio-preparations of Russia, showed negative results either without or with metabolic activity using *Salmonella typhimurium* (TA1534, TA1537, TA1950, TA98, and TA100) as the tester strain [147].

Influence of EBL to root tips of *Allium cepa* was observed for change in growth and mitotic index. It was reported that treatment of 0.005 ppm of 24-EpiBL (low dose) improved the root length and mitotic number in contrast to control by two times. However, treatment of 0.5 ppm (highest dose) inhibited the root length and mitotic index in comparison to control [148]. 24-EBL was isolated and characterized using various chromatographic techniques from *Aegle marmelos* Correa. [Rutaceae]. It was also evaluated for MH (maleic hydrazine) induced antigenotoxicity using the classical aberration assay and it was found that the application of 24-EpiBL significantly reduced the rate of chromosomal aberration due to MH (0.01). The maximum inhibition of 91.8% was observed in 24-EpiBL treatment [149]. Sondhi et al. [150] studied plant derived BRs for their antigenotoxic activity against H_2_O_2_-stimulated DNA injury in human lymphocytes. CS was detected using electrospray mass spectral data from *Centellaasiatica* leaves, a known medicinal herb. Castasterone showed antigenotoxicity in human blood lymphocytes employing comet assay and the 10^−9^ M was found most effectual in suppressing DNA injuries.

### 4.7. Anticholesteromic Action

Phytosterols and their analogues are recognized as suppressors of intestines ability of cholesterol absorption and have been found to lower the total plasma cholesterol and LDL cholesterol levels [151]. BRs being an oxidized form of sterols are assumed to have similar activities as that of plant sterols [152,153]. In plants, the adaptogenic properties of BRs are elucidated in terms of fluidity, membrane permeability, and activities of membrane proteins [154,155]. Recently, BRs have been considered effective agents for cholesterol controlling and arteriosclerosis preventive abilities [156,157].

The cholesterol levels in rat models fed with 24-EpiBL and normal diet was found to have lowered levels of cholesterol by 9–25% at 2–200 µg/kg concentration. When rats were nourished with an elevated cholesterol diet, the application of 2 µg/kg of 24-EpiBL daily for 4 weeks lowered total cholesterol levels by 34% and triglycerides level by 58% when compared to a rat model on normal diet. Additionally, the plasma levels of vitamin E and vitamin A were enhanced by 16% and 35%, respectively, when supplemented with 24-EpiBL. Similar results were observed in rats nourished with an elevated cholesterol diet and daily dose of 20 µg/kg of 24-EpiBL for a period of 4 weeks. Total cholesterol level was lowered by 44%, triglycerides level was lowered by 68%, and low density lipoproteins were found to be lowered by 11% in comparison to the control model [153]. More recently, reports suggested that few nuclear receptors have a pivotal participation in maintenance of blood cholesterol levels in the body [158]. Dietary cholesterol level was found to be lowered in response to activation of nuclear receptor i.e., LXR which induces ABC1 transporter (reverse) of cholesterol that eventually pumps cholesterol out of cellular compartments. The cholesterol lowering activity of 24-EpiBL is also a part of the adaptogenic effects of BRs which are found in plants as well as animals.

A similar pattern of decline in cholesterol level was observed by BRs application [159,160]. A group of 10 people having hypercholesterolemia were given a dose of 15 µg of 24-EpiBl daily for 1 month. Before and after treatment with 24-EpiBL, all the volunteers underwent basic laboratory investigations. There was no difference found in hematological profile and other biochemical parameters, although levels of cholesterol were found to be lowered by 38% and triglycerides were lowered by 41% [159].

### 4.8. Ecdysteroidal Activities

Ecdysteroids (insect molting hormones) control growth, metamorphosis, and reproduction of arthropods and protostomium animals [161,162]. Brassinosteroids share remarkable structural similarity with ecdysteroids, thus, they can affect the growth and development of insects. This led to studies investigating agonistic/antagonistic ecdysteroidal effects of brassinosteroids on insects. Experiments by Richter et al. [163] exhibited that 22S,23S-homobrassinolide had molting delaying effects in *Periplanetaamericana*. Later Spindler et al. [164] reported that 22S, 23S-homobrassinolide and 22S, 23S-homocastasterone had weak competitive affinity for ecdysteroidal receptors from epithelial cell lines of *Chironomustentans.*

Additionally, both phytosteroids exhibited morphological effects similar to 20-ohecdysone such as inhibition of chitin synthesis which also supported agonistic effects of BRs. Antiecdysteroidal effects of BRs and their synthetic precursors were reported by Lehmann et al. [165]. They found inhibition of the action of 20-hydroxyecdysone (biological active ecdysteroid in insects) by competitively binding to ecdysteroidal receptors purified from larvae of *Calliphoravicia.* Ecdysone-like binding affinity for ecdysteroidal receptors purified from the last instar larvae of *Galleria mellonella* was also exhibited by 22S,23S-homocastasterone [166]. In vitro competition binding assay conducted using imaginal wing discs dissected *Spodopteralittoralis* last instar larvae revealed that 24-EpiBL and 24-EpiCS showed 50% competitive binding with labelled ponasterone (A), with no evagination ecdysone analog [162]. Since ecdysteroids are growth regulators of insect larvae, it is likely that antiecdysteroids could be used as a new mode of pest control as they can interfere with growth and development of the target pest [162]. Thus, BRs could be used in modern insecticidal formulations for safer pest management if they have proven antiecdysteroidal effects. Davison later reported that D1 30 pesticide, a BR analogue, retarded larval development and initiated mortality in larvae of *Aedesaegypti* indicating its antiecdysteroidal effect [167]. However, in B_II_ cell assay in *Drosophila melanogaster* a large number of BRs showed neither agonistic nor antagonistic ecdysteroidal activities but exhibited cytotoxicity at higher concentrations [168]. Similarly, 24-epibrassinolide displayed no ecdysteroidal activity in Se4 cell lines of *Spodopteraexiqua* [169]. Cytotoxic effects of BRs are often misinterpreted as ecdysteroidal active [169]. BRs biosynthesis inhibitors may interfere with ecdysteroid biosynthesis owing to their structural similarity Oh et al. [170] observed that triazole type BR biosynthesis inhibitors exhibited larvicidal activity against *A. aegypti*. These studies highlight the need to further explore structural activity relationships between BRs and ecdysteroids that may lead to development of a new class of insecticides.

### 4.9. Anabolic Activities

The ecdysteroidal hormones have been well known for their anabolic and adaptogenic activities [171,172]. Multiple impacts on anabolic activities of rats such as food intake, gain of body weight, enhanced muscle mass of gastrocnemius, and lean body weight were found to be altered in response to application of 28-homoBL (20–60 mg/kg). Application of 28-homoBL results in enhanced physical strength of rats [124]. 24-EpiBL (2–20 mg/kg) was observed to increase the static regulation and physical endurance for swimming in mice [173]. BRs anabolic activities were also used to increase the bull sperm producing efficiency [174]. Other anabolic steroids bind to the intracellular receptor (androgen, this process has ill-effects on humans), whereas, BRs have been found to have a low androgenic activity and do not bind to the androgen receptor [175]. The possible mechanism of BRs action in anabolic and adaptogenic effects on animal systems was demonstrated by Esposito et al. [175]. They suggested that BRs application resulted in activation of the P13K/Akt signaling cascade which further increases Akt phosphorylation in vitro.

## 5. Conclusions

BRs are natural, non-hazardous, nontoxic, eco-friendly, and biosafe plant hormones which are an ideal category of hormones to be used in various therapeutic practices. The screening of BRs for various bioactivities in animal system indicates their potential as a tool in the medical field. BRs have inhibitory effects on multiplication of viruses specifically in human cell lines, at times with a large selectivity index (SI), including cytotoxic consequence in a plethora of cancer cells without affecting normal human cells [127]. A wide range of BRs (both natural and synthetic derivatives) of stigmasterone series have been found to have antiviral and antiherpatic activities. BRs are found to be a novel category of steroids which possess anti-inflammatory activities [115]. More recently, they have been demonstrated to have antiangiogenic, ecdysteroidal, and anabolic activities. Table 2 elaborates various reports on the wide array of therapeutic potential of brassinosteroids. Furthermore, insight into underlying mechanisms of BRs signaling in plants and animal systems is anticipated to contribute to their future in therapeutics for human use.

## Figures and Tables

**Table 1:** Antiviral properties of BRs in response DNA and RNA viruses.

Viruses	(% Inhibition)	
	28-homocastaterone	Brassinolide
Poliovirus type 1 (RNA virus)	85	96
Vesicular stomatitis Virus Indiana strain (RNA virus)	23	100
Herpes simplex virus-1 F strain (tk^+^) (DNA virus)	50	96
Herpes simplex virus-1 B2006 strain (tk^−^) (DNA virus)	35	100
Herpes simplex virus-1 G strain (tk^+^) (DNA virus)	48	98
Junin virus IV _4454_ strain (RNA virus)	79	74
Tacaribe virus TRLV _11573_ strain (RNA virus)	99	55
Pichinde virus AN_3739_ strain (RNA virus)	98	67
Measles virus Brasil/001/91 (RNA virus)	50	100

* Infection was done at a moi (multiplicity of infection) of 1 with several viruses in Vero cells followed by adsorption after 1 h at 37 °C. The cultures were soaked with MM (maintenance medium) or with MM consisting 28-homoCS (40 µM) and brassinolide (1 µM). Supernatants were harvested after 18–24 h of incubation followed by titration through plaque assay. Source: [79].

**Table 2:** Various reports on the wide array of therapeutic potential of brassinosteroids in plants and cell lines (virus, animal, and human models).

S. No.	Class of Compounds	Model System	Health Effects	Mechanism of Action	References
**1.**	Brassinosteroids (28-HomoCS and 24-EpiBL)	Human Breast Cancer Cell Line, Human Prostate Cancer Cell Line	Antiproliferative activity, pro-apoptotic activity, and cell growth inhibitory responses in several human cell lines with no effect on non-tumor cell growth	Cell blockade and apoptosis of both hormone-sensitive and insensitive human breast cancer	[51,65]
**2.**	Brassinosteroids (24-EpiBL and 24-EpiCS)	Human Cancer Cell Line	Antiproliferative, anticancer, antiangiogenic, antiviral, and antibacterial properties in the animal system	Inhibit replication of viruses in confluent with human cell culture, including cytotoxic effects in various types of cancer cells but normal human cells	[134]
**3.**	Brassinosteroids (28-HomoCS)	RNA and DNA Viruses	Antiviral effect against RNA and DNA viruses	Limiting virus protein synthesis and mature viral particle formation	[79]
**4.**	Stigmasterols [(22S,23S)-22,23-dihydroxystigmast-4-en-3-one, (22S,23S)-22,23-dihydroxystigmasta-1,4-dien-3-one]	Murine Macrophage Cell Line	Immuno-modulatory and neuro-protective activity	Blocked HSV-1 induced activation of NFαB by inhibiting its translocation to the nucleus of infected conneal and conjunctival cells in vitro, as well as significantly reduced the secretion of TNF-α infected NHC cells	[95]
**5.**	Brassinosteroids (28-HomoBL and 24-EpiBL)	Human Cancer Cell Line	Anticancer bioactivities in various cell lines i.e., CEM (T-Lymophoblastic Leukemia), A549 (lung carcinoma), MCF-7 (breast carcinoma), LNCaP (prostate cancer) etc.	All these cells were found non-viable in response to 4-fold dilution for 72 h of IC_50_ value of BRs observed from Calcein AM assay	[36]
**6.**	Brassinosteroids (24-EpiBL)	Human Prostate Cancer Cell Line	In vitro antiproliferative effect in the animal cell lines	Cytotoxicity in PC-3 cells activating polyamines catabolic machinery in prostate cancer cells	[66,67]
**7.**	Brassinosteroids (24-EpiBL)	Human Colon Cancer Cell Line	Mitochondria-regulated cell death in colon cancer cells	Upregulation of Foxo3a and protein tryokinase Src p38, after the activation of P13K/AKT	[68]
**8.**	Brassinosteroids (24-EpiBL)	Plant (*Arabidopsis thaliana*)	Amelioration of Turnip crinkle virus infection in *Arabidopsis thaliana*, BAK1 or BKK1 are essential components	Increase in activity of antioxidant enzymes and subsequent gene expression and also lowered photosystem deterioration	[76]
**9.**	Brassinolide	Plant (*Nicotiana benthamiana*)	Increased resistance of *Nicotiana benthamiana* against TMV	BES1/BZR1 suppressed RBOHB-dependent ROS generation regulated by MEK2-SIPK signaling network	[78]
**10.**	Brassinosteroid [(22S,23S)-3beta-bromo-5alpha,22,23-trihydroxystigmastan-6-one]	Vero Cell Line (virus growth)	Antiviral activity against Junin virus (JV)	Detrimental effect on RNA replication of trihydroxystigmastan-6-one and lately result in formation of viral glycoproteins	[82]
**11.**	Brassinosteroids [(22*S*,23*S*)-3β-bromo-5α,22,23-trihydroxystigmastan-6-one]	Herpes Simplex Virus Cell Lines (Vero Cells)	Hampering the herpes simplex virus (HSV) type 1 replication in Vero cells by affecting the later stages of virus multiplication	Inhibition of the expression of HSV antigen and reduced the production of HSV late protein	[93]
**12.**	Brassinosteroids (24-EpiBL)	Plants (*Cucumis sativus*)	24-EpiBL minimized fungal initiated ROS and increased resistance towards *Fusarium*	Reduced *Fusarium* wilt in cucumber and enhanced their antioxidant and phenolic levels in roots	[114]
**13.**	Brassinosteroid analogs [22S, 23S]-3β-bromo-5α, 22, 23-trihydroxystigmastan-6-one, [22S, 23S]-3β-5α, 22, 23-tetrahydroxystigmastan-6-one and [22S, 23S]-5α-fluoro-3β, 22, 23-tetrahydroxystigmastan-6-one	Herpes Simplex Virus Cell Lines (Vero Cells)	Anti-inflammatory activity and inflammation were lowered in three consecutive days	These analogues act against HSV-1 multiplication and exhibited anti-inflammatory potential in vero cells	[52]
**14.**	Brassinosteroid (28-HomoBL Keto-isomers)	Human Conjuctival Cell Lines	Antiglycemic activity in diabetic rats	Improved RBC, WBC, platelets, hemoglobin levels, and reduced cellular damage	[127]
**15.**	Stigmasterone derivatives [22S,23S]-22,23-dihydroxystigmast-4-en-3-one (Compound 1) and [22S,23S]-3β-bromo 5α,22,23-trihydroxystigmastan-6-one (Compound 2)	Human umbilical vein endothelial cells (HUVEC)	Prevents in vivo and in vitro angiogenesis, reduces the VEGF expression and also corneal neovascularization when applied during herpetic stromal keratitis	Retardation in migration of cells migration in human umbilical vein endothelial cells (HUVEC) and capillary tube-like structure formation	[143]
